# Sharing privacy-sensitive access to neuroimaging and genetics data: a review and preliminary validation

**DOI:** 10.3389/fninf.2014.00035

**Published:** 2014-04-07

**Authors:** Anand D. Sarwate, Sergey M. Plis, Jessica A. Turner, Mohammad R. Arbabshirani, Vince D. Calhoun

**Affiliations:** ^1^Department of Electrical and Computer Engineering, Rutgers, The State University of New JerseyPiscataway, NJ, USA; ^2^Mind Research NetworkAlbuquerque, NM, USA; ^3^Department of Psychology and Neuroscience Institute, Georgia State UniversityAtlanta, GA, USA; ^4^Department of Electrical and Computer Engineering, University of New MexicoAlbuquerque, NM, USA

**Keywords:** collaborative research, data sharing, privacy, data integration, neuroimaging

## Abstract

The growth of data sharing initiatives for neuroimaging and genomics represents an exciting opportunity to confront the “small *N*” problem that plagues contemporary neuroimaging studies while further understanding the role genetic markers play in the function of the brain. When it is possible, open data sharing provides the most benefits. However, some data cannot be shared at all due to privacy concerns and/or risk of re-identification. Sharing other data sets is hampered by the proliferation of complex data use agreements (DUAs) which preclude truly automated data mining. These DUAs arise because of concerns about the privacy and confidentiality for subjects; though many do permit direct access to data, they often require a cumbersome approval process that can take months. An alternative approach is to only share data derivatives such as statistical summaries—the challenges here are to reformulate computational methods to quantify the privacy risks associated with sharing the results of those computations. For example, a derived map of gray matter is often as identifiable as a fingerprint. Thus alternative approaches to accessing data are needed. This paper reviews the relevant literature on differential privacy, a framework for measuring and tracking privacy loss in these settings, and demonstrates the feasibility of using this framework to calculate statistics on data distributed at many sites while still providing privacy.

## 1. Introduction

Neuroimaging data has been the subject of many data sharing efforts, from planned large-scale collaborations such as the Alzheimers Disease Neuroimaging Initiative (ADNI) (Jack et al., 2008) and functional biomedical informatics research network (FBIRN) (Potkin and Ford, 2009) (among others) to less-formalized operations such as openfmri.org (Poldrack et al., 2013) and the grass roots functional connectomes project (FCP) with its international extension (INDI) (Mennes et al., 2013). The Frontiers in Neuroinformatics special issue on “Electronic Data Capture, Representation, and Applications in Neuroimaging” in 2012 Turner and Van Horn (2012) included a number of papers on neuroimaging data management systems, several of which provide the research community some access to their data. In many cases, an investigator must agree to a data usage agreements (DUA): they specify who they are, what elements of the data they want, and often what they are planning to do with it. The researcher must agree to abide by arrangements such as not attempting to re-identify the subjects, not re-sharing the data, not developing a commercial application off the data, and so on. These DUAs may be as simple as a one page electronic questionnaire for contact purposes, or a full multi-page form that requires committee review, institutional official review and signatures being faxed back and forth.

The 2012 publication by members of the INCF Task Force on Neuroimaging Datasharing (Poline et al., 2012), specifically on neuroimaging data sharing, reiterated that data should be shared to improve scientific reproducibility and accelerate progress through data re-use. However, the barriers to data sharing that they identified included the well-known problems of motivation (both the ability to get credit for the data collected, as well as the fear of getting “scooped”,) ethical and legal issues, and technical or administrative issues. In many cases, motivation is less of an issue than are the perceived legal and technical issues in keeping an investigator from sharing their data. The perceived legal issues regarding privacy and confidentiality, and protecting the trust that the subject has when they give their time and effort to participate in a study, are what lead to multi-page DUAs.

Neuroimaging is not the only data type whose sharing is hampered by these privacy concerns. Genetic data is perhaps the most contentious to share; the eMERGE consortium worked through a number of issues with large-scale sharing of genetic data, including the usual administrative burdens and ethical concerns (McGuire et al., 2011), and the five sites of the consortium identified numerous inconsistencies across institutional policies due to concerns about ethical and legal protections. It is often easy to re-identify individuals from genetic data; one publication showing re-identification of individuals is even possible from pooled data (Homer et al., 2008), prompting the NIH to remove data from a public repository (Couzin, 2008). Despite the existence of more sophisticated re-identificationattacks (e.g., Schadt et al., 2012), the NIH has not responded by removing the data. One of the most recent efforts re-identified subjects through combining DNA sequences with publicly available, recreational genealogy databases (Gymrek et al., 2013). These publicized privacy breaches make patients rightly concerned about their identifiable health information being shared with unknown parties.

This leads to basically three categories of data that will never be made publicly available for easy access: (1) data that are non-shareable due to obvious re-identification concerns, such as extreme age of the subject or a zip code/disease combination that makes re-identification simple; (2) data that are non-shareable due to more complicated or less obvious concerns, such as genetic data or other data which may be re-identifiable in conjunction with other data not under the investigator's control; and (3) data that are non-shareable due to the local institutional review boards (IRBs) rules or other administrative decisions (e.g., stakeholders in the data collection not allowing sharing). For example, even with broad consent to share the data acquired at the time of data collection, some of the eMERGE sites were required to re-contact the subjects and re-consent prior to sharing within the eMERGE consortium, which can be a permanent show-stopper for some datasets (Ludman et al., 2010).

The first two data types may be shared with an appropriate DUA. But this does not guarantee “easy access;” it can slow down or even prevent research. This is particularly onerous when it is not known if the data being requested are actually useable for the particular analysis the data requestor is planning. For example, it may be impossible to tell how many subjects fit a particular set of criteria without getting access to the full data first (Vinterbo et al., 2012). It is markedly problematic to spend weeks, months, or even years waiting for access to a dataset, only to find out that of the several hundred subjects involved, only a few had usable combinations of data of sufficient quality necessary for one's analysis.

Problems with DUAs only become worse when trying to access data from multiple sites. Because each DUA is different, the administrative burden rapidly becomes unmanageable. In order to enable analyses across multiple sites, one successful approach is to share data derivatives. For example, the ENIGMA consortia pooled together data from many hundreds of local sites and thousands of subjects by providing analysis scripts to local sites and centrally collecting only the output of these scripts (Hilbar et al., 2013). Another example is DataSHIELD (Wolfson et al., 2010), which also uses shared summary measures to perform pooled analysis. These systems are good starting points, but they neither quantify privacy nor provide any guarantees against re-identification. In addition, summary measures are restricted to those that can be computed independently of other data. An analysis using ENIGMA cannot iterate among sites to compute results informed by the data as a whole. However, by allowing data holders to maintain control over access, such an approach does allow for more privacy protections at the cost of additional labor in implementing and updating a distributed architecture.

The ENIGMA approach is consistent with the *differential privacy* framework (Dwork et al., 2006), a strong notion of privacy which measures the risk of sharing the results of computations on private data. This quantification allows data holders to track overall risk, thereby allowing local sites to “opt-in” to analyses based on their own privacy concerns. However, in the differential privacy model, the computation is *randomized*—algorithms introduce noise to protect privacy, thereby making the computation less accurate. However, if protecting privacy permits sharing data derivatives, then aggregating private computations across many sites may lead to a benefit; even though each local computation is less accurate (to protect privacy), the “large N” benefit from many sites allowing access will still result in a more accurate computation.

The system we envision is a research consortium in which sites allow differentially-private computations on their data without requiring an individual DUA for each site. The data stays at each site, but the private data derivatives can be exchanged and aggregated to achieve better performance. In this paper we survey some of the relevant literature on differential privacy to clarify if and how it could help provide useful privacy protections in conjunction with distributed statistical analyses of neuroimaging data. The default situation is no data sharing: each site can only learn from its own data. We performed an experiment on neuroimages from a study to see if we could predict patients with schizophrenia from healthy control subjects. Protecting privacy permits a pooled analysis; without the privacy protections, each site would have to use its own data to learn a predictor. Our experiments show that by gathering differentially private classifiers learned from multiple sites, an aggregator can create a classifier that significant outperforms that which could be learned at a single site. This demonstrates the potential of differential privacy: sharing access to data derivatives (the classifiers) improves overall accuracy.

Many important research questions can be answered by the kind of large-scale neuroinformatics analyses that we envision.

- Regression is a fundamental statistical task. Regressing covariates such as age, diagnosis status, or response to a treatment against structure and function in certain brain regions (voxels in an image) is simple but can lead to important findings. For example, in examining the ability to aggregate structural imaging across different datasets (Fennema-Notestine et al., 2007) used the regression of age against brain volumes as a validity test. Age also affects resting state measures, as Allen et al. (2011) demonstrated on an aggregated dataset of 603 healthy subjects combined across multiple studies within an individual institution that had a commitment to data sharing and had minimal concerns regarding re-identification of the data. In that study, because privacy and confidentiality requirements that limited access to the full data, the logistics of extracting and organizing the data took the better part of a year (personal communication from the authors). In such a setting, asking a quick question such as whether age interacts with brain structure differently in healthy patients versus patients with a rare disorder would be impossible without submitting the project for IRB approval. This process can take months or even years and cost hundreds of dollars, whereas the analysis takes less than a day and may produce negative findings. We need a framework that facilitates access to data on the fly for such straightforward but fundamental analyses.
- The re-use of genetic data has been facilitated by dbGAP, NIH's repository for sharing genome-wide scan datasets, gene expression datasets, methylation datasets, and other genomic measures. The data need to be easily accessible for combined analysis for identification or confirmation of risk genes. The success of the Psychiatric Genomic Consortium in finding confirmed risk genes of schizophrenia after almost 5 years of aggregating datasets supports these goals of making every dataset re-usable (Ripke et al., 2013). While dbGAP has been a resounding success, it has its drawbacks. Finding the data can be a bit daunting, as often phenotype data is made available separately from the genetic data. For example, the PREDICT-HD Huntington's disease study rolled out a year before the genetic data. DbGAP's sharing requirements are driven by the need to ensure the data are handled appropriately and the subjects' confidentiality and privacy are protected; requesting a dataset entails both the PI and their institutional official signing an agreement as well as a review by the study designate. This process must be completed prior to access being granted or denied. As before, this precludes any exploratory analyses to identify particular needs, such as determining how many subjects have the all the required phenotype measures.
- The success of multimodal data integration in the analysis of brain structure/function (Plis et al., 2010; Bießmann et al., 2013; Bridwell et al., 2013; Schelenz et al., 2013), imaging/genetics (Liu et al., 2012; Chen et al., 2013; van Erp et al., 2013), and EEG/fMRI (Bridwell et al., 2013; Schelenz et al., 2013) shows that with enough data, we can go further than simple univariate linear models. For example, we can try to find combinations of features which predict the development of a disorder, response to various treatments, or relapse. With more limited data there has been some success in reproducing diagnostic classifications (Arbabshirani et al., 2013; Deshpande et al., 2013), and identifying coherent subgroupings within disorders which may have different genetic underpinnings (Girirajan et al., 2013). With combinations of imaging, genetic, and clinical profiles from thousands of subjects across autism, schizophrenia, and bipolar disorder, for example, we could aim to identify more clearly the areas of overlap and distinction, and what combinations of both static features and dynamic trajectories in the feature space identify clinically relevant clusters of subjects who may be symptomatically ambiguous.

## 2. Privacy models and differential privacy

There are several different conceptual approaches to defining privacy in scenarios involving data sharing and computation. One approach is to create *de-identified* data; these methods take a database of records corresponding to individuals and create a *sanitized database* for use by the public or another party. Such approaches are used in official statistics and other settings—a survey of different privacy models can be found in Fung et al. (2010), and a survey of privacy technologies in a medical informatics context in Jiang et al. (2013). These approaches differ in how they define privacy and what guarantees they make with respect to this definition. For example, *k*-anonymity (Sweeney, 2002) quantifies privacy for a particular individual *i* with data *x_i_* (for example, age and zip code) in terms of the number of other individuals whose data is also equal to *x_i_*. Algorithms for guaranteeing *k*-anonymity manipulate data values (e.g., by reporting age ranges instead of exact ages) to enforce that each individual's record is identical to at least *k* other individuals.

A different conceptual approach to defining privacy is to try and quantify the change in the risk of re-identification as a result of publishing a function of the data. This differs from data sanitizing methods in two important respects. Firstly, privacy is a property of an algorithm operating on the data, rather an a property of the sanitized data—this is the difference between *semantic* and *syntactic* privacy. Secondly, it can be applied to systems which do not share data itself but instead share data derivatives (functions of the data). The recently proposed ϵ-differential privacy model (Dwork et al., 2006) quantifies privacy in terms of risk; it bounds the likelihood that someone can re-infer the data of an individual. Algorithms that guarantee differential privacy are *randomized*—they manipulate the data values (e.g., by adding noise) to bound the risk.

Finally, some authors define privacy in terms of data security and say that a data sharing system is private if it satisfies certain cryptographic properties. The most common of these models is secure multiparty computation (SMC) (Lindell and Pinkas, 2009), in which multiple parties can collaborate to compute a function of their data without leaking information about their private data to others. The guarantees are cryptographic in nature, and do not assess the re-inference or re-identification problem. For example, in a protocol to compute the maximum element across all parties, a successful execution would reveal the maximum. A secondary issue is developing practical systems to work on neuroinformatics data. Some progress has been made in this direction (Sadeghi et al., 2010; Huang et al., 2011; Nikolaenko et al., 2013), and it is conceivable that in a few years SMC will be implemented in real distributed systems.

### 2.1. Privacy technologies for data sharing

As discussed earlier, there are many scenarios in which sharing raw data is either difficult or impossible—strict DUAs, obvious re-identification issues, difficulties in assessing re-identifiability, and IRB or other policy rules. Similar privacy challenges exists in the secondary use of clinical data (National Research Council, 1997). In many medical research contexts, there has been a shift toward sharing *anonymized* data. The Health Insurance Portability and Accountability Act (HIPAA) privacy rule (45 CFR Part 160 and Subparts A and E of Part 164) allows the sharing of data as long as the data is de-identified. However, many approaches to anonymizing or “sanitizing” data sets (Sweeney, 2002; Li et al., 2007; Machanavajjhala et al., 2007; Xiao and Tao, 2007; Malin, 2008) are subject to attacks (Sweeney, 1997; Ganta et al., 2008; Narayanan and Shmatikov, 2008) that use public data to compromise privacy.

When data sharing itself is precluded, methods such as *k*-anonymity (Sweeney, 2002), *l*-diversity (Machanavajjhala et al., 2007), *t*-closeness (Li et al., 2007), and *m*-invariance (Xiao and Tao, 2007) are no longer appropriate, since they deal with constructing private or sanitized versions of the data itself. In such situations we would want to construct data access *systems* in which data holders do not share the data itself but instead provide an interface to the data that allows certain pre-specified computations to be performed on that data. The data holder can then specify the granularity of access it is willing to grant subject to its policy constraints.

In this model of *interactive data access*, the software that controls the interface to the raw data acts as a “curator” that screens queries from outsiders. Each data holder can then specify the level of access which it will provide to outsiders. For example, a medical center may allow researchers to access summaries of clinical data for the purposes of exploratory analysis; a researcher can assess the feasibility of doing a study using existing records and then file a proposal with the IRB to access the real data (Murphy and Chueh, 2002; Murphy et al., 2006; Lowe et al., 2009; Vinterbo et al., 2012). In the neuroinformatics context, data holders may allow outside users to receive a histogram of average activity levels for regions of a certain size.

Being able to track the privacy risks in such an interactive system allows data holders to match access levels with local policy constraints. The key to privacy tracking is *quantification*—for each query or access to the data, a certain amount of information is “leaked” about the underlying data. With a sufficient number of queries it is theoretically possible to reconstruct the data (Dinur and Nissim, 2003), so the system should be designed to mitigate this threat and allow the data holders to “retire” data which has been accessed too many times.

### 2.2. Differential privacy

A user of the database containing private information may wish to apply a *query* or algorithm to the data. For example, they may wish to know the histogram of activity levels in a certain brain region for patients with a specified mutation. Because the answer to this query is of much lower dimension than a record in the database, it is tempting to regard disclosing the answer as not incurring a privacy risk. A important observation of Dinur and Nissim (2003) was that an adversary posing such queries may be able to reconstruct the entire database from the answers to multiple simple queries. The *differential privacy* model was introduced shortly thereafter, and has been adopted widely in the machine learning and data mining communities. The survey by Dwork and Smith (2009) covers much of the earlier theoretical work, and Sarwate and Chaudhuri (2013) review some works relevant to signal processing and machine learning. In the basic model, the database is modeled as a collection of *N* individuals' data records 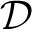 = (*x*_1_, *x*_2_, …, *x_N_*), where *x_j_* is the data for individual *j*. For example, *x_j_* may be the MRI data associated to individual *j* together with information about mutations in certain genes for that individual.

An even simpler example is to estimate the mean activity in a certain region, so each *x_j_* is simply a scalar which represented the measured activity of individual *j*. Let us call this desired algorithm Alg. Without any privacy constraint, the data curator would simply apply Alg to the data 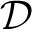 to produce an output *h* = Alg(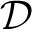). However, in many cases the output *h* could compromise the privacy of the data and unfettered queries could lead to reidentification of an individual.

Under differential privacy, the curator applies an approximation PrivAlg to the data instead of Alg. The approximation PrivAlg is *randomized*—the randomness of the algorithm ensures that an observer of the output will have a difficult time re-identifying any individual in the database. More formally, PrivAlg(·) provides ϵ-differential privacy if for any subset of outputs 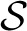,

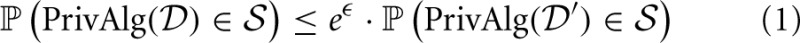



for any two databases 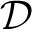 and 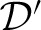 differing in a single individual. Here ℙ(·) is the probability over the randomness in the algorithm. It provides (ϵ, δ)-differential privacy if

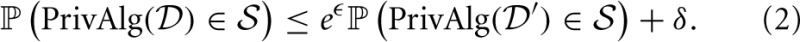



The guarantee that differential privacy makes is that the distribution of the output of PrivAlg does not change too much, regardless of whether any individual *x_j_* is in the database or not. In particular, an adversary observing the output of PrivAlg and knowing all of the data of individuals in 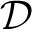 ∩ 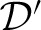 common to both 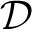 and 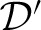 will still be uncertain of the remaining individual's data. Since this holds for any two databases which differ in one data point, each individual in the database is guaranteed of this protection. More specifically, the parameters ϵ and δ control the tradeoff between the false-alarm (Type I) and missed-detection (Type II) errors for an adversary trying to make a test between 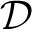 and 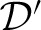 (see Oh and Viswanath, 2013 for a discussion).

Returning to our example of estimating the mean, the desired algorithm Alg is simply the sample mean of the *m* data points, so 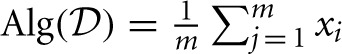. The algorithm Alg itself does not provide privacy because output is deterministic: the distribution of Alg(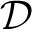) is a point mass exactly at the average. If we change one data point to form, say 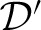 = (*x*_1_, *x*_2_, …, *x*_*m*−1_, *x*′_*m*_), then Alg(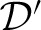) ≠ Alg(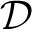) and the only way Equation (1) can hold is if ϵ = ∞. One form of a private algorithm is to add noise to the average (Dwork et al., 2006). A differentially private algorithm is 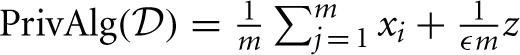, where *z* has a Laplace distribution with unit variance. The Laplace distribution is a popular choice, but there are many other distributions which can also guarantee differential privacy and may be better in some settings (Geng and Viswanath, 2012, 2013). For more general functions beyond averages, Gupte and Sundararajan (2010) and Ghosh et al. (2012) showed that in some cases we can find optimal mechanisms, while Nissim and Brenner (2010) show that this optimality may not be possible in general.

Although some variations on these basic definition have been proposed in the literature (Chaudhuri and Mishra, 2006; Rastogi et al., 2009; Kifer and Machanavajjhala, 2011), most of the literature focuses on ϵ- or (ϵ, δ)-differential privacy. Problems that have been studied in the literature range from statistical estimation (Smith, 2011; Kifer et al., 2012; Smith and Thakurta, 2013), to cover more complex data processing algorithms such as real-time signal processing (Fan and Xiong, 2012; Le Ny and Pappas, 2012a,b), classification (Chaudhuri et al., 2011; Rubinstein et al., 2012; Zhang et al., 2012b; Jain and Thakurta, 2014), online learning (Jain et al., 2012; Thakurta and Smith, 2013), dimensionality reduction (Hardt et al., 2012; Chaudhuri et al., 2013), graph estimation (Karwa et al., 2011; Kasiviswanathan et al., 2013), and auction design (Ghosh and Roth, 2011). The preceding citations are far from exhaustive, and new papers on differential privacy appear each month as methods and algorithms become more mature.

There are two properties of differential privacy which enable the kind of *privacy quantification* that we need in shared data-access scenarios. The first property is *post-processing invariance*: the output of an ϵ-differentially private algorithm PrivAlg maintains the same privacy guarantee—if *ĥ* = PrivAlg(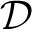), then the output of any function *g*(*ĥ*) applied to *ĥ* is also ϵ-differentially private, provided *g*(·) doesn't depend on the data. This means that once the data curator has guaranteed ϵ-differential privacy for some computation, it need not track how the output is used in further processing. The second feature is *composition*—if we run two algorithms PrivAlg_1_ and PrivAlg_2_ on data 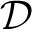 with privacy guarantees ϵ_1_ and ϵ_2_, then combined they have privacy risk at most ϵ_1_ + ϵ_2_. In some cases these composition guarantees can be improved (Dwork et al., 2010; Oh and Viswanath, 2013).

### 2.3. Differentially private algorithms

A central challenge in the use of differentially private algorithms is that by using randomization to protect privacy, the corresponding accuracy, or *utility*, of the result is diminished. We contend that the potential for a much larger sample size through data sharing makes this tradeoff worthwhile. In this section we discuss some of the differentially private methods for statistics and machine learning that have been developed in order to help balance privacy and utility in data analyses.

Differentially private algorithms have been developed for a number of important fundamental tasks in basic statistics and machine learning. Wasserman and Zhou (2010) put the differential privacy framework in a general statistical setting, and Smith (2011) studied point estimation, showing that many statistical quantities can be estimated with differential privacy with similar statistical efficiency. Duchi et al. (2012, 2013) studied a different version of *local* privacy and showed that requiring privacy essentially entails an increase in the sample size. Since differential privacy is related to the stability of estimators under changes in the data, Dwork and Lei (2009) and Lei (2011) used tools from robust statistics to design differentially private estimators. Williams and McSherry (2010) studied connections to probabilistic inference. More recently, Kifer et al. (2012) proposed methods for high-dimensional regression and Smith and Thakurta (2013) developed a novel variable selection method based on the LASSO.

One approach to designing estimators is the sample-and-aggregate (Nissim et al., 2007; Smith, 2011; Kifer et al., 2012), which uses subsampling of the data to build more robust estimators. This approach was applied to problems in sparse linear regression (Kifer et al., 2012), and in particular to analyze the LASSO (Smith and Thakurta, 2013) under the slightly weaker definition of (ϵ, δ)-differential privacy. There are several works which address convex optimization approaches to statistical model selection and machine learning under differential privacy (Chaudhuri et al., 2011; Kifer et al., 2012; Rubinstein et al., 2012; Zhang et al., 2012b) that encompass popular methods such as logistic regression, support vector machines, and other machine learning methods. Practical kernel-based methods for learning with differential privacy are still in their infancy (Chaudhuri et al., 2011; Jain and Thakurta, 2013).

### 2.4. Challenges for differential privacy

In addition to the theoretical and algorithmic developments, some authors have started trying to build end-to-end differentially private analysis toolkits and platforms. The query language PINQ (McSherry, 2010) was the first tool that allowed people to write differentially-private data-analysis programs that guarantee differential privacy, and has been used to write methods for a number of tasks, including network analyses (McSherry and Mahajan, 2010). Fuzz (Reed and Pierce, 2010) is a functional programming language that also guarantees differential privacy. At the systems level, AIRAVAT (Roy et al., 2010) is a differentially private version of MapReduce and GUPT (Mohan et al., 2012) uses the sample-and-aggregate framework to run general statistical algorithms such as *k*-means. One of the lessons from these implementations is that building a differentially private *system* involves keeping track of every data access—each access can leak some privacy—and systems can be vulnerable to attack from adversarial queries (Haeberlen et al., 2011).

A central challenge in designing differentially private algorithms for practical systems is setting the privacy risk level ϵ. In some cases, ϵ must be chosen to be quite large in order to produce useful results—such a case was studied in earlier work by Machanavajjhala et al. (2008) in the context of publishing differentially private statistics about commute times. On the other side, choosing a small value of ϵ may result in adding too much noise to allow useful analysis. To implement a real system, it is necessary to do a proper evaluation of the impact of ϵ on the utility of the results. Ultimately, the setting of ϵ is a policy decision that is informed by the privacy-utility tradeoff.

There are several difficulties with implementing existing methods “off the shelf” in the neuroinformatics context. Neuroimaging data is often continuous-valued. Much of the work on differential privacy has focused on discrete data, and algorithms for continuous data are still being investigated theoretically (Sarwate and Chaudhuri, 2013). In this paper we adapt existing algorithms, but there is a need to develop methods specifically designed for neuroimage analyses. In particular, images are high-dimensional signals, and differentially private version of algorithms such as PCA may perform poorly as the data dimension increases (Chaudhuri et al., 2013). Some methods do exist that exploit structural properties such as sparsity (Hardt and Roth, 2012, 2013), but there has been insufficient empirical investigation of these methods. Developing low-dimensional representations of the data (perhaps depending on the task) can help mitigate this.

Finally, neuroimaging datasets may contain few individuals. While the signal from each individual may be quite rich, the number of individuals in a single dataset may be small. Since privacy affects the statistical efficiency of estimators, we must develop distributed algorithms that can leverage the properties of datasets at many locations while protecting the privacy of the data at each. Small sample sizes present difficulties for statistical inference without privacy—the hope is that the larger sample size from sharing will improve statistical inference despite the impact of privacy considerations. We illustrate this in the next section.

## 3. Applying differential privacy in neuroinformatics

In the absence of a substitute for individual DUAs, sites are left to perform statistical analyses on their own data. Our proposal is to have sites participate in consortium in which they share differentially private data derivatives, removing the need for individual DUAs. Differential privacy worsens the quality of a statistical estimate at a single site because it introduces extra noise. However, because we can share the results of differentially private computations at different sites, we can reduce the impact of the noise from privacy. This larger effective sample size can give better estimates than are available at a single site, even with privacy. We illustrate this idea with two examples. The first is a simple problem of estimating the mean from noisy samples, and the second is an example of a classification problem.

### 3.1. Estimating a mean

Perhaps the most fundamental statistical problem is estimating the mean of a variable. Suppose that we have *N* sites, each with *m* different samples of an unknown effect:

(3) $$x_{i,j}=\mu +z_{i,j}\;i=1,2,\ldots ,N,\;j=1,2,\ldots ,m,$$

where μ is an unknown mean, and *z*_*i,j*_ is normally distributed noise with zero mean and unit variance. Each site can compute its local sample mean:

(4) $${\bar{X}}_{i}=\frac{1}{m}\sum_{j=1}^{m}x_{i,j}=\mu +\frac{1}{m}\sum_{j=1}^{m}z_{i,j}.$$

The sample mean *X*_*i*_ is a an estimate of μ which has an error that is normally distributed with zero mean and variance $\frac{1}{m}$. Thus a single site can estimate μ to within variance $\frac{1}{m}$. A simple ϵ-differentially private estimate of μ is

(5) $${\tilde{X}}_{i}=\frac{1}{m}\sum_{j=1}^{m}x_{i,j}+\frac{1}{\epsilon m}w_{i},$$

where *w*_*i*_ is a Laplace random variable with unit variance. Thus a single site can make a differentially private estimate of μ with error variance $\frac{1}{m}+\frac{1}{{\left(\epsilon m\right)}^{2}}$. Now turning to the *N* sites, we can form an overall estimate using the differentially private local estimates:

(6) $$\bar{X}=\frac{1}{N}\sum_{i=1}^{N}{\tilde{X}}_{i}=\mu +\frac{1}{mN}\sum_{i=1}^{N}\sum_{j=1}^{m}x_{i,j}+\frac{1}{\epsilon mN}\sum_{i=1}^{N}w_{i}.$$

This is an estimate of μ with variance $\frac{1}{mN}+\frac{1}{{\left(\epsilon m\right)}^{2}N}$.

The data sharing solution results in a lower error compared to the local non-private solution whenever $\frac{1}{m}>\frac{1}{mN}+\frac{1}{{\left(\epsilon m\right)}^{2}N}$, or

$$N>1+\frac{1}{\epsilon^{2}m}.$$

As the number of sites increases, we can support additional privacy at local nodes (ϵ can decrease) while achieving superior statistical performance over learning at a single site *without privacy*.

### 3.2. Classification

We now turn to a more complicated example of differentially private classification that shows how a public data set can be enhanced by information from differentially private analyses of additional data sets. In particular, suppose there are *N* sites with private data and 1 site with a publicly available dataset. Suppose private site *i* has *m_i_* data points {($\vec{x}$_*i,j*_, *y_i,j_*) : *j* = 1, 2, …, *m_i_*}, where each $\vec{x}$_*i,j*_ ∈ ℝ^*d*^ is a *d*-dimensional vector of numbers representing features of the *j*-th individual at site *i*, and *y*_*i,j*_ ∈ {−1, 1} is a label for that individual. For example, the data could be activity levels in certain voxels and the label could indicate a disease state. Each site can learn a classifier on its own local data by solving the following minimization problem.

(7) $${\vec{w}}_{i}=\underset{\vec{w}\in {\mathbb{R}}^{d}}{\arg\min}\sum_{j=1}^{m_{i}}\ell (y_{i,j}{\vec{w}}^{⊤}{\vec{x}}_{i,j})+\frac{\lambda }{2}\|\vec{w}{\|}^{2},$$

where ℓ(·) is a loss function. This framework includes many popular algorithms: for the support vector machine (SVM) ℓ(*z*) = max(0, 1 − *z*) and for logisticregression ℓ(*z*) = log(1 + *e*^−*z*^).

Because the data at each site might be limited, they may benefit from producing differentially private versions ${\vec{w}}_{i}$ and then combining those with the public data to produce a better overall classifier. That is, leveraging many noisy classifiers may give better results than any ${\vec{w}}_{i}$ on its own. The method we propose is to train *N* differentially private classifiers using the objective perturbation method applied to the Huberized support vector machine (see Chaudhuri et al., 2011 for details). In this procedure, the local sites minimize a perturbed version of the classifier given in Equation (7). Let ${\vec{w}}_{i}$ be the differentially private classifier produced by site *i*.

Suppose the public data set has *m*_0_ points {($\vec{x}$_0,*j*_, *y*_0,*j*_) : *j* = 1, 2, …, *m*_0_}. We compute a new data set {(${\vec{u}}_{0,j}$, *y*_0,*j*_): *j* = 1, 2, …, *m*_0_} where ${\vec{u}}_{0,j}$ is an *N*-dimensional vector whose *i*-th component is equal to ${\vec{w}}_{i}^{⊤}{\vec{x}}_{0,j}$. Thus ${\vec{u}}_{0,j}$ is the vector of “soft” predictions of the *N* differentially private classifiers produced by the private sites. The public site then uses logistic regression to train a new classifier:

(8) $${\vec{w}}_{0}=\underset{\vec{w}\in {\mathbb{R}}^{d}}{\arg\min}\sum_{j=1}^{m_{0}}\log(1+e^{-y_{0,j}{\vec{w}}^{⊤}u_{0,j}})+\frac{\lambda }{2}\|\vec{w}{\|}^{2}.$$

**Figure 1:**
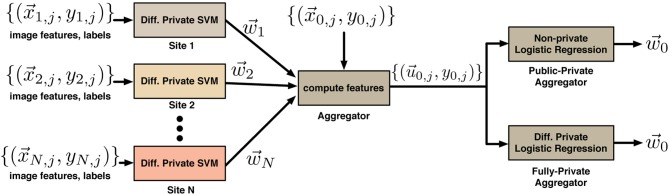
**System for differentially private classifier aggregation from many sites**. The *N* sites each train a classifier on their local data to learn vectors {${\vec{w}}_{i}$}. These are used by an aggregator to compute new features for its own data set. The aggregator can learn a classifier using its own data using a non-private algorithm (if its data is public) or a differentially private algorithm (if its data is private).

This procedure is illustrated in Figure 1. The overall classification system produced by this procedure consists of the classifiers {${\vec{w}}_{i}$ : *i* = 0, 1, …, *N*}. To classify a new point $\vec{x}$ ∈ ℝ^*d*^, the system computes $\vec{u}=({\vec{w}}_{1}^{⊤}\vec{x},{\vec{w}}_{2}^{⊤}\vec{x},\ldots ,{\vec{w}}_{N}^{⊤}\vec{x})$ and then predicts the label ŷ = sign($\left({\vec{w}}_{0}^{⊤}\vec{u}\right)$). In the setting where the public site has more data, training a classifier on pairs $\left(\vec{u},\vec{x}\right)$ could also work better.

**Figure 2:**
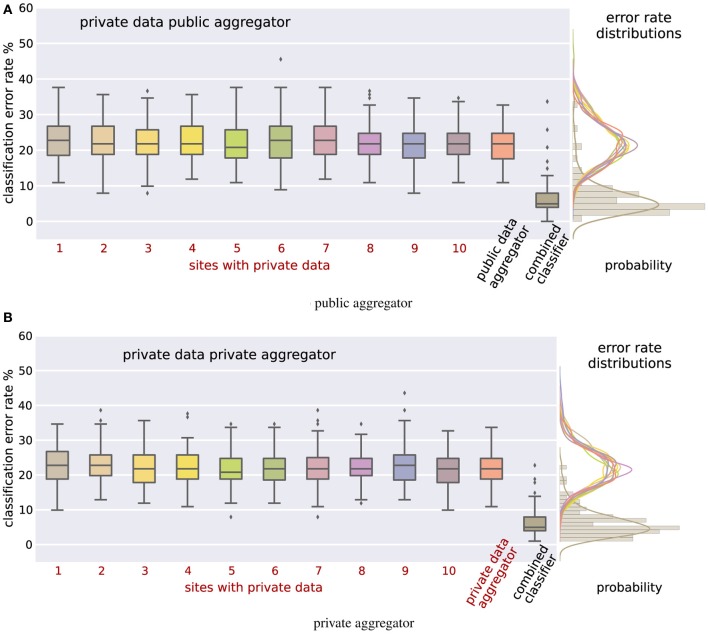
**Classification error rates for the mixed private-public case (A) and the fully-private case (B)**. In both cases the combined differentially private classifier performs significantly better than the individual classifiers. The difference is statistically significant even after Bonferroni correction (to account for multiple sites) with corrected *p*-values below 1.8 × 10^−33^. Results thus motivate the use of differential privacy for sharing of brain imaging and genetic data to enable quick access to data which is either hard to access for logical reasons or not available for open sharing at all.

We can distinguish between two cases here—in the *public-private* case, described above, the classifier in Equation (8) uses differentially private classifiers from each of the *N* sites on public data, so the overall algorithm is differentially private with respect to the private data at the *N* sites. In the *fully-private* case, the data at the (N + 1)-th site is also private. In this case we can replace Equation (8) with a differentially private logistic regression method (Chaudhuri et al., 2011) to obtain a classifier which is differentially private with respect to the data at all *N* + 1 sites. Note, although we assign the role of constructing the overall two-level classifier to either the public-data site or one of the private sites in the real use-case no actual orchestrating of the process is required. It is convenient for the purposes of the demonstration (and without loss of generality) to treat a pre-selected site as an aggregator, which we do in the experiments below. Figure 2. can only be interpreted if we are consistent with the site that does the aggregation. However, all that needs to be done for the whole system to work is for the *N* (or *N* + 1 in the fully private case) private sites compute and publish their classifiers ${\vec{w}}_{i}$. Then in the public data case, anyone (even entities with no data), can construct and train a classifier by simply downloading the publicly available dataset and following the above-described procedure. This could be one of the sites with the private data as well. When no public data is available the second level classifier can be only computed by one of the private-data sites (or each one of them) and later published online to be useful even for entities with insufficient data. In both cases, the final classifier (or classifiers) is based on a larger data pool that is available to any single site.

From the perspective of differential privacy it is important to note that the only information that each site releases about its data is the separating hyperplane vector ${\vec{w}}_{i}$ and it does so only once. Considering privacy as a resource a site would want to minimize the loss of this resource. For that, a single release of information in our scheme is better that multiple exchanges in any of the iterative approaches (e.g., Gabay and Mercier, 1976; Zhang et al., 2012a).

We implemented the above system on a neuroimaging dataset (structural MRI scans) with *N* = 10 private sites. We combined data from four separate schizophrenia studies conducted at Johns Hopkins University (JHU), the Maryland Psychiatric Research Center (MPRC), the Institute of Psychiatry, London, UK (IOP), and the Western Psychiatric Institute and Clinic at the University of Pittsburgh (WPIC) (see Meda et al., 2008). The sample comprised 198 schizophrenia patients and 191 matched healthy controls (Meda et al., 2008). Our implementation relies on the differentially private SVM and logistic regression as described by Chaudhuri et al. (2011) and implementation available online^1^. The differentially private Hubertized SVM in our implementation used regularization parameter λ = 0.01, privacy parameter ϵ = 10, and the Huber constant *h* = 0.5, while parameters for differentially private logistic regression were set to λ = 0.01 and ϵ = 10 (for details see Chaudhuri et al., 2011). The quality of classification depends heavily on the quality of features; because distributed and differentially private feature learning algorithms are still under development, for the purposes of this example we assume features are given. To learn the features for this demonstration we used a restricted Boltzmann machine (RBM) (Hinton, 2000) with 50 sigmoidal hidden units. For training we have employed an implementation from Nitish Srivastava^2^. We have used *L*_1_-regularization of the feature matrix *W*(λǁ*W*ǁ_1_) (λ = 0.1) and 50% dropout to encourage sparse features and effectively handle segmented gray matter images of 60465 voxels each. The learning rate parameter was set to 0.01. The weights were updated using the truncated Gibbs sampling method called contrastive divergence (CD) with a single sampling step (CD-1). Further information on RBM model can be found in Hinton (2000) and Hinton et al. (2006). After the RBM was trained we activated all 50 hidden units on each subject's MRI producing a 50 dimensional dataset. Note, no manual feature selection was involved as each and every feature was used. Using these features we repeated the following procedure 100 times:

1. Split the complete set of 389 subjects into class-balanced training and test sets comprising 70% (272 subjects) and 30% (117 subjects) of the data, respectively. The training set was split into *N* + 1 = 11 class-balanced subsets (sites) of 24 or 25 subjects each.
2. Train a differentially private SVM on *N* = 10 of these subsets independently (sites with private data).
3. Transform the data of the 11th subset (aggregator) using the trained SVM classifiers (as described above).
4. Train both a differentially private classifier (fully-private) and a standard logistic regression classifier (public-use) on the transformed dataset (combined classifier).
5. Compute the individual error rates on the test set for each of the *N* = 10 sites. Compute the error rates of a (differentially private) SVM trained on the data of 11th dataset and the aggregate classifier in Equation (8) that uses differentially private results from all of the sites.

The results that we obtained in this procedure are summarized in Figure 2 for the mixed private-public (Figure 2A) as well as the fully-private (Figure 2B) cases. The 10 sites with private data all have base-line classification error rates of a little over 20%, indicating the relative difficulty of this classification task and highlighting the effect of the noise added for differential privacy. That is, on their own, each site would only be able to learn with that level of accuracy. The distribution of the error rates across experiments is given to the right. The last column of each figure shows the error rate of the combined classifier; Figure 2A shows the results for a public aggregator, and Figure 2B for the private aggregator. In both cases the error rate of the aggregated classifier is around 5%, which is a significant improvement over a single site. Additionally, the distribution of the error of the combined classifier is more tightly concentrated about its mean. To quantify the significance of the improvement we performed 2-sample *t*-tests for the distribution of the error rates of the combined classifier against error rate distributions of classifiers produced at individual sites. The largest Bonferroni corrected *p*-value was 1.8 × 10^−33^. The experiments clearly show the benefits of sharing the results of differentially private computations over simply using the data at a single site. Even though the classifier that each site shares is a noisy version of what they could learn privately and thus less accurate, aggregating noisy classifiers produces at multiple sites dramatically lowers the resulting error.

## 4. Discussion

Data sharing interfaces must take into account the realities of neuroimaging studies—current efforts have been very focused on the data structures and ability to query, retrieve and share complex and multi-modal datasets, usually under a fixed model of centralized warehousing, archiving, and privacy restrictions. There has been a remarkable lack of focus on the very important issues surrounding the lack of DUAs in older studies and also the privacy challenges which are growing as more data becomes available and predictive machine learning becomes more common.

We must consider several interlocking aspects when choosing a data sharing framework and the technology to enable it. Neuroimaging and genetics data present significant unique challenges for privacy. Firstly, this kind of data is very different from that considered by many works on privacy—images and sequence data are very high-dimensional and highly identifiable, which may set limits on what we expect to be achievable. Secondly, we must determine the data sharing structure—how is data being shared, and to whom. Institutional data holders may allow other institutions, individual researchers, or the public to access their data. The structure of the arrangement can inform which privacy technology is appropriate (Jiang et al., 2013). Thirdly, almost all privacy-preserving data sharing and data mining technologies are still under active research development and are not at the level of commercially deployed security technologies such as encryption for e-Commerce. A privacy-preserving computation model should be coupled with a legal and policy framework that allows enforcement in the case of privacy breaches. In our proposed model, sites can participate in a consortium in which only differentially private data derivatives are shared. By sharing access to the data, rather than the data itself, we mitigate the current proliferation of individually-generated DUAs, by allowing local data holders to maintain more control.

There are a number of challenges in building robust and scalable data sharing systems for neuroinformatics. On the policy side, standards and best practices should be established for data sharing within and across research consortia. For example, one major challenge is attribution and proper crediting for data used in large-scale studies. On the technology side, building federated data sharing systems requires additional fault-tolerance, security, and more sophisticated role-management than is typically found in the research environment. As noted by Haeberlen et al. (2011) implementing a differentially private system introduces additional security challenges without stricter access controls. Assigning different trust levels for different users (Vinterbo et al., 2012), managing privacy budgets, and other data governance policy issues can become quite complicated with differential privacy. On the statistical side, we must extend techniques from meta-analyses to interpret statistics computed from data sampled under heterogenous protocols. However, we believe these challenges can be overcome so that researchers can more effectively collaborate and learn from larger populations.

## Funding

This work was supported by the National Institutes of Health via awards NIMH U01MH097435 (Lei Wang, PI) to Jessica A. Turner and NIBIB R01EB005846 and COBRE 5P20RR021938/P20GM103472 to Vince D. Calhoun.

### Conflict of interest statement

The authors declare that the research was conducted in the absence of any commercial or financial relationships that could be construed as a potential conflict of interest.

## Footnotes

^1^http://cseweb.ucsd.edu/~kamalika/code/dperm/

^2^https://github.com/nitishsrivastava/deepnet
